# The Medicinal Treatment of Cancer

**Published:** 1904-12-10

**Authors:** 


### THE MEDICINAL TREATMENT OF CANCER.

In an article on "Hypodermic Medication in Inoperable Cancer," Dr. Jno. A. Shaw-Mackenzie [1] relates a number of instances in which he has obtained good results by the use of medicines introduced either into the intra-cellular or intra-muscular tissues. His earlier experiences related to iodopin—a combination of iodine and sesame oil—in cases of uterine fibroids. The solution was introduced into the intra-cellular tissue of the buttock in doses of 3 to 10 c.c. In no case did any local or general inconvenience follow the injections. In a number of instances the symptoms were completely relieved with more or less shrinkage of the tumour, and in two cases the fibroid practically disappeared. For cases of inoperable cancer two remedies were employed — namely, chian turpentine and soap solution. The former, it will be remembered, was some twenty years or more ago strongly advised by the late Professor Clay, of Birmingham. Other observers failed to obtain good results, and the method fell into disuse. It is now suggested that failure may have been due to the administration of the remedy in a form which did not admit of absorption, and, in support of this, it is claimed that when given as pills the drug may be passed per rectum in an undigested condition. Further, it is said that the best results followed the use of the chian turpentine prescribed as an emulsion. As a further improvement, Dr. Shaw-Mackenzie suggests the injection of a 20 per cent. solution of chian turpentine in olive oil deeply into the subcutaneous tissues of the buttock. Of this he commences with a dose of 5 minims, increasing by 5 minims on alternate days to a maximum of 60 minims. The site of the injection should be sterilised by some antiseptic solution and then anæsthetised by the use of ice or other local anæsthetic. A stout needle, preferably of irido-platinum, is necessary. One of the cases related in the paper was that of an old soldier with a large malignant mass fixed to the structures on the right side of the neck and ulcerated in several places. Under the administration of chian turpentine pain ceased, the offensive discharge was checked, and the tumour shrank greatly in size. In a second case the growth was of pelvic origin and was producing considerable disturbance of the stomach and bladder. Here also the tumour rapidly lessened under the chian-turpentine treatment. In reference to the use of soap solution particulars of cases are promised in a future paper, but the results are described as sufficient to justify the hope that we may perhaps be able "one day to control malignant growths by hypodermic medication."

Dr. G. Cooke Adams,[2] in an elaborate article on cancer in Australia, insists upon the influence of syphilis as a cause strongly predisposing the tissues to malignant disease; and to the diminution of syphilis and the avoidance of the various causes of prolonged local irritation he looks for the successful preventive treatment of cancer. As a means of arresting malignant growths he advocates the use of a special eucalyptus oil, to which he has given the name "mulyptol." Classifying 55 cases of cancer (mostly of the breast and uterus) in which operation had been refused, and in which the treatment he advises had been applied under his personal supervision for from six to twelve months, he states that, though inquiries were made over two to four years after the treatment was discontinued, in not one single instance was recurrence reported. The remedy was both given internally and applied locally. The mulyptol was ordered either as an emulsion or in gelatine capsules, the dose being five minims every four hours after food for the first week, and gradually increased to 10-minim doses by the end of the second month. The oil was also applied by means of inunction, being rubbed in over the axillary and abdominal regions night and morning, and inhalations with steam were administered twice daily. In the case of cancer of the breast the remedy was applied as an emulsion and by vapour as well as in the form of dressings. For the first 10 months as a rule the improvement was slight, yet by the end of the fourth month it is said that in most cases "all signs of the growth and malignant condition had disappeared." The local treatment in uterine cancer consisted in scraping the ulcerated surface of the cervix, painting the part every alernate day with pure mulyptol, douching with a solution of the drug, packing the vagina with medicated gauze, etc. Treatment was persevered with from three to six months after all signs of malignant disease had disappeared. Dr. Adams is strongly convinced of the great value of the special variety of eucalyptus oil which he describes, and he urges that a fair trial should be given to it in all inoperable cases of malignant disease.

[1] Medical Press and Circular, Oct. 19, 1904. [2] Lancet, Feb. 20, 1904.

